# Intramuscular Vaccination With the HSV-1(VC2) Live-Attenuated Vaccine Strain Confers Protection Against Viral Ocular Immunopathogenesis Associated With γδT Cell Intracorneal Infiltration

**DOI:** 10.3389/fimmu.2021.789454

**Published:** 2021-11-15

**Authors:** Rafiq Nabi, Andrew C. Lewin, Therese M. Collantes, Vladimir N. Chouljenko, Konstantin G. Kousoulas

**Affiliations:** ^1^ Department of Pathobiological Science, Louisiana State University School of Veterinary Medicine, Baton Rouge, LA, United States; ^2^ Department of Veterinary Clinical Sciences, Louisiana State University School of Veterinary Medicine, Baton Rouge, LA, United States

**Keywords:** herpes simplex, gamma delta T cells, immunopathogenesis, ocular infection, herpes keratitis

## Abstract

Herpes simplex virus type-1 (HSV-1) ocular infection is one of the leading causes of infectious blindness in developed countries. The resultant herpetic keratitis (HK) is caused by an exacerbated reaction of the adaptive immune response that persists beyond virus clearance causing substantial damage to the cornea. Intramuscular immunization of mice with the HSV-1(VC2) live-attenuated vaccine strain has been shown to protect mice against lethal ocular challenge. Herein, we show that following ocular challenge, VC2 vaccinated animals control ocular immunopathogenesis in the absence of neutralizing antibodies on ocular surfaces. Ocular protection is associated with enhanced intracorneal infiltration of γδ T cells compared to mock-vaccinated animals. The observed γδ T cellular infiltration was inversely proportional to the infiltration of neutrophils, the latter associated with exacerbated tissue damage. Inhibition of T cell migration into ocular tissues by the S1P receptors agonist FTY720 produced significant ocular disease in vaccinated mice and marked increase in neutrophil infiltration. These results indicate that ocular challenge of mice immunized with the VC2 vaccine induce a unique ocular mucosal response that leads into the infiltration of γδ T cells resulting in the amelioration of infection-associated immunopathogenesis.

## Introduction

Herpetic Keratitis (HK) induced by herpes simplex virus type 1 (HSV-1) ocular infection is a leading cause of infectious blindness. It is estimated that 50-90% of the world population is infected with HSV-1 (1–3). Primary infection of HSV-1 targets mucosal regions such as the oral lining and skin (4). After the establishment of latency in the trigeminal ganglion (TG), HSV-1 can reactivate due to various environmental factors and physiological stress, leading to HK (5, 6). HK is widely considered to be an immune-mediated condition where uncontrolled inflammatory events continue to damage the cornea during and after the resolution of infection (7).

The use of animal models such as mice and rabbits is well-established in ocular HSV-1 research (6). Data from these animal models suggest that after initial reactivation from the TG, the virus travels to the ocular surface in an anterograde manner (8, 9). The presence of the virus on the ocular surface activates several innate immune pathways (10) that lead to inflammation and subsequent tissue damage. It has been suggested that vaccine-induced immunity can reduce HSV-1 induced HK in animal models (11–17). However, there is currently no approved vaccine for clinical use. Previously, we reported that intramuscular immunization (IM) with the live-attenuated HSV-1 vaccine (VC2) confers complete protection against a lethal HSV-1 challenge and ocular immunopathogenesis (18–20).

The HSV-1 (VC2) vaccine strain derived from the laboratory attenuated parental HSV-1 (F) strain specifies glycoprotein K (gK) having a 39 amino-terminal deletion of glycoprotein K (gK). This amino acid deletion has been shown to prevent entry into cells *via* fusion of the viral membrane with cellular plasma membranes including neuronal axons. In contrast, the virus replicates efficiently in a variety of cells, because it can enter through endocytosis (21–23). In addition, the VC2 vaccine strain has a deletion in the amino terminus of the membrane protein UL20 that interacts with the carboxyl terminus of glycoprotein B (gB). The UL20 protein functions as a heterodimer with gK to modulate the fusogenic properties of gB and both gK. Thus, the combined effect of the gK/UL20 mutations provide a unique safety feature to the VC2 virus, since it cannot infect neurons *via* neuronal axons and establish latency (24–30). HSV-1 gK has an important role in virus-induced corneal scaring (CS). Specifically, immunization with gK or overexpression of gK caused exacerbated virus-induced CS. gK-induced CS depends on gK binding to signal peptidase (SPP), while its binding partner UL20 binds GODZ (DHHC3) that are involved in gK-induced pathology (31–35).

HSV-1 infection of the corneal epithelium induces a cascade of antiviral innate and downstream adaptive immune responses (10). Innate responses are mediated by neutrophils, plasmacytoid dendritic cells (pDCs), natural killer (NK) cells and macrophages (MQ), which have direct and indirect antiviral functions (36–39). These innate responses possess potent antiviral activity, however, exacerbated responses can cause tissue damage. This phenomenon is particularly true for tissue damage caused by neutrophil accumulation in two separate waves (39, 40) despite their beneficial role in viral clearance (39, 41). Innate immune responses are followed by the adaptive immune response, which mainly involves CD4+T and CD8+T cells appearing as early as 3 days post-infection exhibiting potent antiviral activities that limit viral spread (42, 43). However, an exacerbated adaptive CD4+T cell response and to a lesser extent a CD8+T cell response can lead to corneal epithelium damage and herpetic stromal disease (44). Thus, a balanced immune response at ocular surfaces is needed to control excessive inflammation and tissue damage, particularly in the case of herpes ocular infections.

Herein, we report that intramuscular immunization of mice with the VC2 vaccine strain, but not with UV-inactivated VC2 or mock-vaccination, induced ocular protection against lethal ocular challenge with the human ocular and highly pathogenic clinical strain HSV-1(McKrae). This tissue-specific protection was associated with γδT cell infiltration with reduced neutrophil accumulation compared to groups received mock and inactivated vaccine. Further, observations suggest that this infiltrated population is not HSV-1 specific memory population although their presence is required to control immunopathogenesis induced by the infection.

## Materials and Methods

### Cells and Viruses

African green monkey (Vero) cells were maintained in complete Dulbecco’s Modified Eagle Medium (DMEM) (Gibco) supplemented with 10% Fetal Calf Serum (FBS) (ThermoFisher). VC2 was constructed as described previously (30). Briefly, the VC2 recombinant virus was constructed utilizing the two-step Double-Red Recombination protocol using the HSV-1(F) viral genome cloned as a bacterial artificial chromosome (BAC). The virus was cultivated in Vero cells. HSV-1((McKrae) was a gift by the late Dr. James Hill (Louisiana State University Health Sciences, Center, New Orleans, LA).

### Vaccination Schedule and Challenge

Female Balb/CJ mice (8-10-week-old) were purchased from Jackson Laboratories, (Bar Harbor, ME USA) and were housed in the Louisiana State University School of Veterinary Medicine (LSU-SVM) ABSL2 facility. A prime-boost vaccination strategy was used. For prime, 100µl of vaccine (10^7^ PFU in DMEM) were injected intramuscularly into the right hind leg followed by a booster dose into the left hind leg 21 days later. Mock vaccinated animals received PBS. All animals were challenged 21 days or 8 months after the last vaccination with a lethal dose (10^6^/eye) of HSV-1 (McKrae). For challenge, animals were anesthetized, and a linear partial epithelial corneal debridement was performed with a 27G needle before 10µl of HSV-1 (McKrae) was applied to the ocular surface. Animals were observed daily for clinical signs of disease and euthanized as described in the IACUC euthanasia criteria.

### Ocular Scoring

Ocular scoring of mice was performed by Dr. Andrew Lewing, a board-certified veterinary ophthalmologist (ACL) according to a modified established ocular disease scoring system (45). Briefly, this scoring system provides objective categorization of corneal opacity, corneal neovascularization, corneal ulceration and ocular discharge [see **Table 1** for the scoring system]. All examinations were performed following induction of a light plane of anesthesia using inhaled isoflurane in oxygen using a handheld biomicroscope (Kowa, SL-17). Normal animals were assigned a score of 0 per eye, and animals with ocular disease were assigned a score of up to 8 per eye, for a maximum possible score of 16 per animal at each time point. All animals were confirmed to be normal with a score of 0 for each eye prior to challenge and were scored again 5 days post infection.

**Table 1:** Ocular scoring system of mice.

Category	Score	Description
Ocular Discharge	0	Normal
	1	Mild ocular discharge – usually watery/clear, covering skin and fur surrounding eye
	2	Severe ocular discharge – usually yellow/tenacious, covering skin and fur surrounding eye. Palpebral fissure may be initially sealed due to copious discharge.
Corneal Opacity (scar/edema)	0	Normal
	1	Mild corneal opacity due to edema and/or corneal fibrosis. Can typically make out intraocular detail using slit beam
	2	Severe corneal opacity due to edema and/or corneal fibrosis. Cannot typically visualize intraocular detail using slit beam
Corneal Neovascularization	0	Normal
	1	Mild corneal neovascularization. Small number (1–2) of thin blood vessels, extending into cornea from limbus
	2	Severe corneal neovascularization. Large number (3+) or blood vessels of varying diameter, extending into cornea from limbus
Corneal Ulceration	0	Normal
	1	Visible corneal ulceration using slit lamp, extending into corneal stroma
	2	Visible corneal perforation using slit lamp

Adapted from Eaton et al., 2017 (45).

### Tissue Processing and Flow Cytometry

The whole mouse eye was collected in PBS following euthanasia, mince and incubated with collagenase in HBSS buffer for 2 hours. The homogenized solution was passed through a 70µm filter to prepare a single cell solution. Mandibular lymph nodes (mLN) were collected and processed through a 70µm filter to prepare a single cell solution. A pre-titrated antibody mixture was incubated for 30 minutes at 4°C, washed and fixed with 2% paraformaldehyde. The next day, samples were analyzed using the BD FACS-Aria equipment and data was processed using FCS Express 7. The anti-mouse antibodies used for flow cytometry were; CD45-APC Vio770, Ly6G-PE CF594, CD3-PerCP-ef710, CD4-FITC, CD8a-BV650, γδTCR-APC, MHCii-BV711, CD11C-PE. CD64-BV605, CD49b-BV421, CD19-BV786, CD44-BV711 and CD62L-BV421. Gating strategy is presented in **Supplementary Figure S1**.

### Immunofluorescence Microscopy and Detection of HSV-RNA Using RNAScope

Following euthanasia, eyes were immediately fixed using 10% formalin for 3 days and processed in the Histology Core Facility of LSU-SVM. For IFM and RNAScope, 5µm thick Formalin-Fixed Paraffin-Embedded (FFPE) sections were prepared on glass slides. To detect viral replication, the HSV-1 UL-48 RNA was used as the target gene with the Probe-V-HSV-1-UL48-C3 purchased from Advanced Cell Diagnostics. The RNAScope assay was performed according to ACDBio guidelines using the Opal 620 dye (Akoya Biosciences) as the substrate. Following the RNAScope assay, the slides were blocked with 10%FBS and incubated overnight with rabbit polyclonal anti-HSV-1 (Dako). The following day slides were washed and anti-rabbit FITC was used as the secondary antibody. Next, the background was reduced with the TrueView auto fluorescence kit (Vectorlabs). After adding mounting media with DAPI, slides were visualized with a Zeiss observer Z1 inverse microscope.

### IFM on Frozen Sections

Whole mouse eyes were collected after euthanasia and immediately frozen using OCT in liquid nitrogen. Samples were stored at -80°C until processing. For microscopy, 8µm thick frozen sections were prepared on glass slides using a cryostat. Tissue sections were fixed briefly for 1 minute using 2% paraformaldehyde at room temperature (RT). After blocking with 5% FBS for 1 hour, slides were incubated with fluorophore-conjugated primary antibody overnight at 40°C. Slides were then washed, fixed and mounting media was added. Images were captured with a Zeiss observer Z1 microscope. The antibodies used for IFM were: Anti-γδTCR-AF488 (GL3), anti-LY6G-AF594 (1A8) and Alexa Fluor 594 anti-mouse CD31 (MEEC13.3) (Biolegend. Inc.) and LYVE-1 AlexaFluor488 (ALY7) (Thermofisher, Inc).

### Bromodeoxyuridine Proliferation Assay

BrdU labeling reagent (Invitrogen, cat# 000103) was injected (1ml/Kg) intraperitoneally (IP) on the day before challenge and administered every day following infection for 4 days. At day 5, animals were euthanized, cells were stained and fixed as mentioned above. Cells were then permeabilized using permeabilization buffer (eBioscience) and incorporated BrdU was stained using Rb-anti-BrdU primary antibodies and aRb-AlexaFluor488 secondary antibodies. Data was captured using a BD FACS Aria Flow cytometer and calculated as a percentage of cells positive for BrdU within a specific population.

### Quantification of Viral Shedding

At 5 days post infection (dpi), viral shedding on the ocular surface was determined using plaque assay. For virus collection, a sterile cotton swab soaked in DMEM was swabbed gently on the ocular surface and collected in a 1.5 ml Eppendorf tube containing DMEM. Samples were stored at -80°C. For virus quantification, a viral plaque assay was performed using Vero cells. Vero monolayers were prepared in 12 well tissue culture plates and incubated with 10-fold dilutions of each sample at room temperature (RT) for 1 hour with shaking. Cells were subsequently washed with complete medium and incubated with complete medium with 1% methyl cellulose for 3 days at 37°C with 5% CO_2_ for viral plaque formation. Next, plates were washed, fixed using 10% formalin and stained with crystal violet. Viral plaques were counted using a light microscope and calculated according to the dilution factor.

### Neutralization Assay

Mouse tears were collected using a sterile cotton swab and placed in a 1.5 ml Eppendorf tube containing DMEM. Each sample represents a pool of 5 animals. Approximately 100 PFU of HSV-1 (McKrae) was mixed with 0.5 ml DMEM containing tears and incubated at 37°C for 1 hour for neutralization. After incubation, the mixture was placed on Vero monolayers on 12 well plates and incubated at room temperature with shaking. The plates were subsequently washed and incubated with complete medium with 1% methyl cellulose for 3 days at 37^0^C with 5% CO_2_ for viral plaque formation.

### qPCR

Following challenge with HSV-1(McKrae), TGs were collected at the time of euthanasia and kept frozen until analysis. On the day of analysis, TGs were thawed, and total DNA was collected using the Qiagen Blood and Tissue kit per manufacturer’s instructions. HSV-1 glycoprotein D (gD) was used as the target gene for quantification using the following primer-probe mixtures purchased from IDT; gD FP – 5^’^-GTCCGGAAACAACCCTACAA-3^’^, gD RP – 5^’^-GCATTCGGTGTACTCCATGA-3^’^, and qPCR Probe – PrimeTime 5’ 6-FAM™/ZEN™/3’ 5^’^-TTGGTTTCGGATGGGAGGCAACT-3^’^ IB^®^FQ. For qPCR, the Prime time Gene Expression Master Mix (IDT) was used according to the manufacturer’s instructions and the reaction was run using the 7900HT Fast Real-Time PCR System with the 384-Well Block Module. TGs from naïve animals were used to set cut-off values and the gD G-block (IDT) was used to create a standard curve.

### FTY720 Treatment

To reduce T cell infiltration in the mouse eyes following infection, FTY720 (Millipore-Sigma) was applied topically to ocular surfaces. FTY720 was dissolved in water at 10 mg/mL. One drop (approx. 10 μl) of this solution was then applied twice daily to the ocular surface one day before infection and continued until 10 DPI. Uninfected naïve mice were treated similarly as controls.

### Multiplex Immunoassay

Whole mouse eyes were collected following euthanasia and immediately frozen using liquid nitrogen. On the day of the assay the eyes were pulverized using a mortar and pestle while frozen. The resulted homogenized tissue was weighed and dissolved in Tris-based lysis buffer (Thermofisher) supplemented with protease inhibitor for total protein extraction. For detecting cytokine and chemokine, the Cytokine & Chemokine Convenience 26-Plex Mouse ProcartaPlex™ Panel 1 (Thermofisher) was used according to manufactures instructions and data was acquired using the Bioplex200 equipment.

### Statistical Analysis

Statistical analysis was performed using GraphPad Prism 9 software. Survival analysis was performed using the log-rank test. For analysis between three groups one-way ANOVA and Kruskal-Wallis test were performed. To compare results between two groups, the Mann-Whitney test was utilized. The statistical significance level was set at p = 0.05.

## Results

### The HV-1(F) VC2 Live-Attenuated Vaccine Generates Robust and Durable Immune Responses in Mice

**Figure 1:**
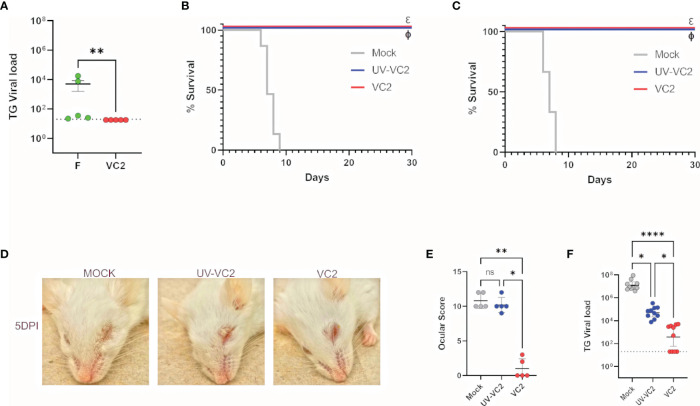
Safety and protective efficacy of VC2 immunization in Balb/CJ mice against HSV-1 (McKrae strain) ocular challenge. **(A)** Mouse eyes were scarified and infected with HSV-1(F) or VC2 viruses at 10^6^ PFU per eye and viral loads in TGs were quantified by qPCR at 20 days post infection. **(B)** Kaplan-Meir survival curves of mice immunized with either, VC2, UV-irradiated VC2 or mock-immunized and challenged with HSV-1 (McKrae strain) at 21 days post booster immunization. The experiment was duplicated with n=10/group each time. **(C)** Kaplan-Meir survival curves as with **(B)** challenged with HSV-1 (McKrae strain) at 8 months post booster immunization, n=10/group. **(D)** Representative pictures of animals in each group at 5 DPI. **(E)** Ocular scores following challenge at 5 DPI. **(F)** Quantification of TG viral load following euthanasia. *<P=0.05, ****<P=0.0001 by One-Way ANOVA. *<P=0.05, **<P=0.005, ****<P=0.0001. ns, Non-Significant.

The VC2 vaccine strain specifies the amino-terminal deletion of 39 amino acids of glycoprotein K (gK), which has been shown to prevent entry into neuronal axons as well as fusion of the virus with cellular plasma membranes, while the virus replicates efficiently because it can enter epithelial and other cells through endocytosis (21–23). To confirm that the VC2 vaccine strain that contains the gK and the UL20 amino terminal deletions cannot infect neuronal endings, travel to the TG and establish latency, mouse corneas of naïve mice were infected with 10^6^ PFU per eye after mild-scarification with either the HSV-1(F) parental wild-type virus or the VC2 vaccine strain. Both the parental HSV-1(F) and VC2 viruses were avirulent, since none of the mice succumbed to the infection. Two weeks post infection, the amount of viral DNA in the TGs was quantified by quantitative PCR. HSV-1(F) but not VC2 viral DNA was detected in ganglionic tissues indicating that VC2 was unable to reach the TGs and establish latency (**Figure 1A**).

Previously, we reported that VC2 intramuscular vaccination of mice generates robust protection against lethal ocular HSV-1 (McKrae) challenge (18). To assess whether this protective immune response is virus replication-dependent and sustained over time, we immunized mice with VC2 or Ultraviolet (UV)-inactivated VC2 and challenged the mice ocularly with HSV-1 (McKrae) at 21 days or 8 months after the booster immunization. VC2 vaccinated mice were fully protected at both time points (**Figures 1B, C**), and there were no apparent ocular and/or systemic clinical disease symptoms observed (**Figure 1D**). In contrast, mice immunized with the UV-inactivated VC2 succumbed to the HSV-1 (McKrae) within 5 DPI and significant ocular damage was noted characterized by ocular inflammation and cornea damage (**Figures 1D, E**). Determination of the relative number of viral genomes in ganglionic tissues by qPCR revealed that a significantly higher level of viral DNA was present in the TG of UV-VC2 vaccinated animals compared to those vaccinated with VC2 (**Figure 1F**). This data suggests IM immunization with live attenuated VC2 generates a robust protection compared to vaccination with inactivated VC2 virions.

### Absence of Neutralizing Antibody on Ocular Surfaces of VC2-Vaccinated Mice

**Figure 2:**
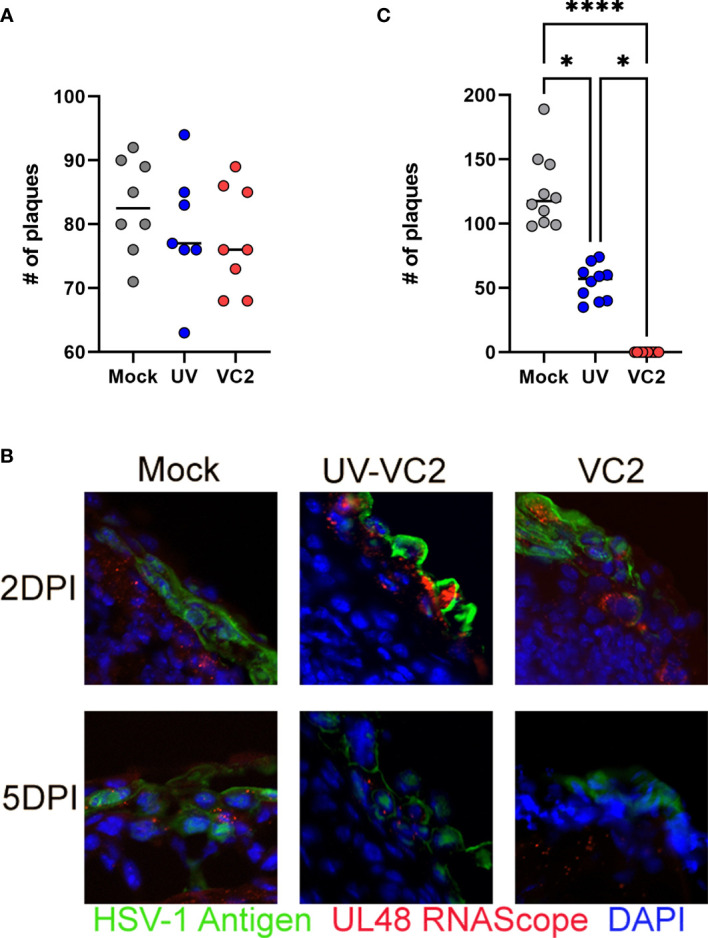
Neutralizing antibodies in mouse tears and post-challenge viral replication in ocular tissues. **(A)** Neutralization of virus after the incubation with tear fluids. **(B)** Viral replication in ocular tissues following challenge at 2- & 5DPI detected by IFM and RNAScope assays. Detection of viral antigens (green) HSV-1 UL48 transcripts (red). **(C)** Viral shedding on ocular surfaces quantified by plaque assay at 5DPI. *<P=0.05, ****<P=0.0001 by One-Way ANOVA.

Previously, we showed that VC2 prime-boost intramuscular immunization induces a strong systemic neutralizing antibody response (18). To assess the contribution of neutralizing antibody in the observed ocular protection against HSV-1 (McKrae) infection, tears from vaccinated mice with either the VC2 vaccine, the UV-inactivated VC2, or mock-vaccinated mice prior to challenge were tested for the presence of neutralizing antibody using a plaque reduction neutralizing assay. Tears from VC2 and VC2-inactivated vaccinated animals did not have higher neutralizing ability compared to mock-vaccinated animals (**Figure 2A**), suggesting the absence of strong neutralizing antibody activity at ocular surfaces. Detection of viral antigens on FFPE sections by indirect immunofluorescence of ocular tissues with anti-HSV-1 polyclonal antibody revealed the presence of viral antigens on ocular surfaces at 2- and 5 DPI in all groups of animals (**Figure 2B**, green). In addition, transcription of the HSV-1 UL48 gene was detected in all ocular tissues using the RNAScope assay (**Figure 2B**, red dots). These results indicated productive infection and viral replication in all groups for at least 5DPI. However, at 5DPI viral shedding in VC2-vaccinated animals were undetectable compared to the mock and UV-VC2-vaccinated mice (**Figure 2C**). This data suggests that despite productive infection and ocular viral replication, the VC2-vaccinated animals experienced robust ocular mucosal responses that significantly reduced viral infection and resultant immunopathogenesis in the absence of neutralizing activity.

### Ocular Protection Is Associated With γδT Cell Infiltration

**Figure 3:**
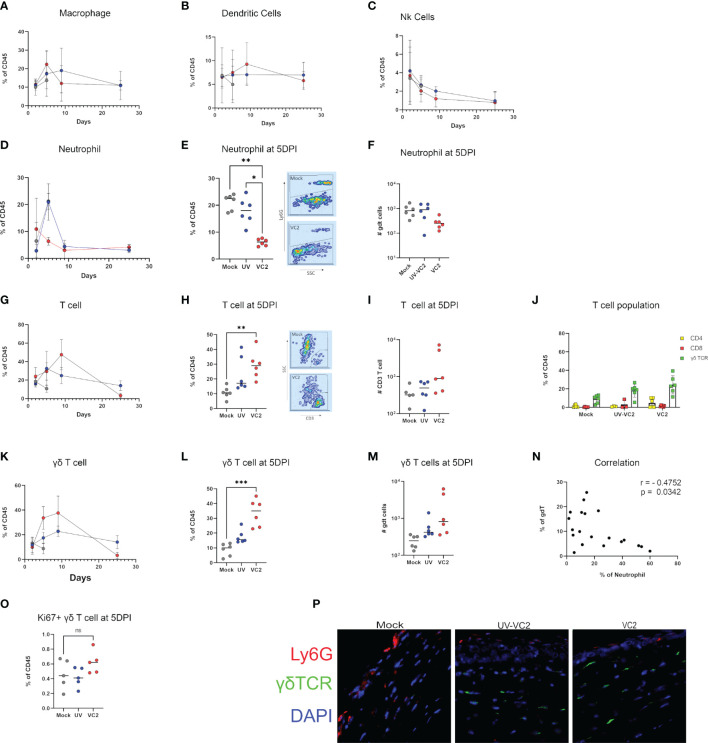
Phenotype of cellular infiltrates in ocular tissues following viral challenge. Flow cytometric analysis for cellular infiltration for: **(A)** Macrophage. **(B)** Dendritic cells. **(C)** NK cells over time. **(D)** Percentage of neutrophil accumulation over time. **(E, F)** Percentage and absolute count of neutrophils at 5DPI. **(G)** Percentage of T cell accumulation over time. **(H, I)** Percentage and absolute count of T cells. **(J)** Phenotype of T cells in individual groups. γδT cell accumulation over time **(K)**, percentage **(L)** and absolute count **(M)** at 5DPI. **(N)** Spearman's rank correlation between γδT cell and neutrophil accumulation among all groups. **(O)** Ki67+ γδT cell in cornea. **(P)** IFM for the presence of neutrophils and γδT using OCT section. **<P=0.005, ***<P=0.001 using One-Way ANOVA, n=6/group/timepoint.

To assess the extent of cell-mediated immunity at ocular surfaces following infection with HSV-1 (McKrae), immune cellular infiltration was evaluated at 2-, 5- and 9 DPI. No detectable differences were noted for macrophages, dendritic cells and NK cells infiltration at any time points (**Figures 3A–C**). A large neutrophil influx was noted following infection in mock-vaccinated animals at 5DPI (**Figure 1E**). In contrast, VC2 vaccinated animals showed significantly lower neutrophil counts at 5DPI (**Figures 3D–F**). A significantly higher T cell infiltration was noted in VC2, but not in mock-vaccinated mice at 5DPI (**Figures 3G–I**). Further analysis revealed that the majority of the infiltrating T cells in all groups of mice expressed the gamma-delta (γδ) TCR, while a relatively small population of CD4+, CD8+ and double negative TCR T cells were also present (**Figures 3J–M**). These results show that γδT cells were the dominant population of immune cells in vaccinated animals, while neutrophils were the major immune cell infiltrate in mock-vaccinated animals at 5DPI. Non-parametric Spearman correlation among all groups of mice revealed a negative correlation between γδT cells and neutrophil accumulation in corneas at 5DPI among all groups (**Figure 3N**). The infiltrating γδT cell numbers peaked at 9 DPI and eventually dropped to basal levels at 25 DPI (after the resolution of ocular pathogenesis (**Figure 3K**). Importantly, the UV-VC2 vaccinated group of mice had lower γδT cell accumulation and high neutrophil accumulation at 5DPI in comparison to the VC2-vaccinated mice that correlated with the observed elevated ocular disease scores in UV-VC2 *versus* VC2-vaccinated mice (**Figures 1E**, **3E, H**).

To address whether the presence of γδT cells following infection in VC2-vaccinated animals is the result of local proliferation or infiltration, a flow cytometry analysis for Ki67, a marker for cellular proliferation, was performed. Following infection, there was no significant increase of Ki67^+^ γδT cells in VC2-vaccinated animals (**Figure 3O**) suggesting that the presence of γδT cell is due to the increased infiltration rather than local proliferation of resident γδT cells. In addition, γδT cells were detected by indirect immunofluorescence on OCT sections of ocular tissues in the corneal stroma of vaccinated animals with little to no neutrophil presence (**Figure 3P**, right-most panel). In contrast, a high number of neutrophils were detected in the corneal stroma and epithelium in mock-vaccinated animals, while low numbers of γδT cells were also present (**Figure 3P**, left-most panel). Taken together, this data suggests VC2 vaccinated animals recruit γδT cells following infection at the ocular surface and that increased neutrophil migration is prevented.

### Increase Lymphoangiogenesis and Reduced Neovascularization Is Associated With γδT Cell Infiltration

**Figure 4:**
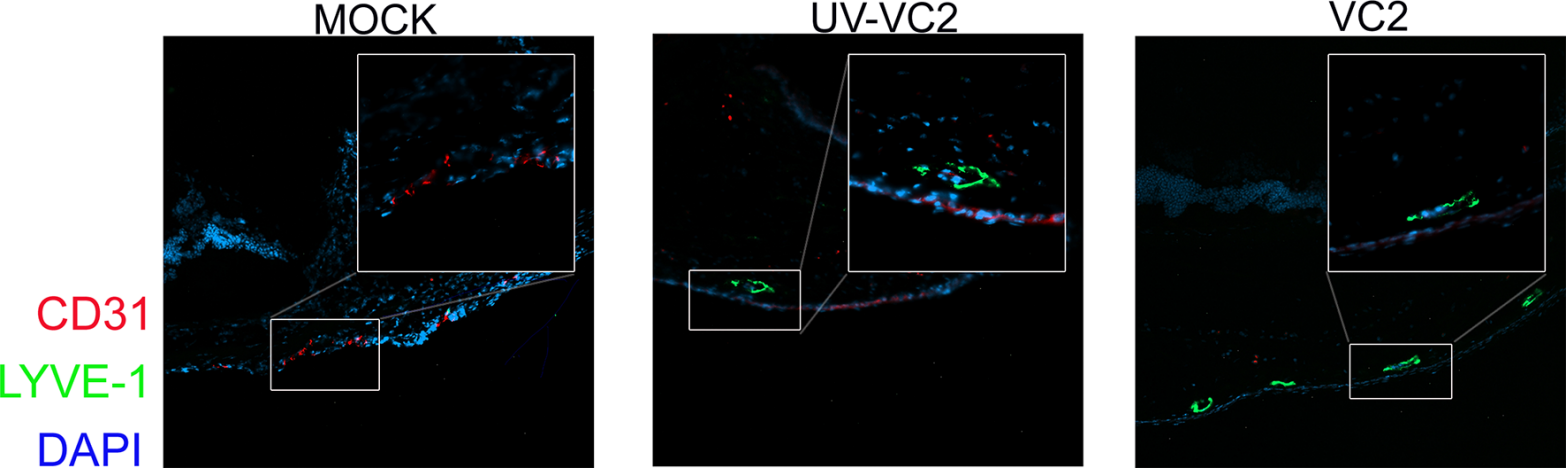
Neovascularization and lymph-angiogenesis in ocular tissues. IFM on OCT sections was used to detect the presence of neovascularization (CD31-red) and lymph-angiogenesis (LYE-1 green).

HSV-1 infection induces neovascularization following infection (46), and infiltrating immune cells may prolong neovascularization by secreting pro-angiogenic growth factors. This neovascularization is likely to be the source of increased levels of corneal neutrophils (47). To assess whether the VC2-vaccination affects neovascularization, OCT sections were stained for LYVE-1, a marker of lymph-angiogenesis (48) and CD31, a marker of angiogenesis (49). HSV-1 infection in mock-vaccinated animals had high levels of CD31 expression (**Figure 4**, left panel) as also described previously (46, 49). In contrast, VC2-vaccinated animals exhibited strong reactivity with the anti-LYVE-1 antibody, while little or no CD31 expression was detectable in the cornea (**Figure 4**, right panel). This data suggests that neovascularization is not responsible for the higher infiltrating γδT cells in VC2 immunized mice.

### Increased Levels of IL-4 and IL-22 Is Associated With Protection

**Figure 5:**
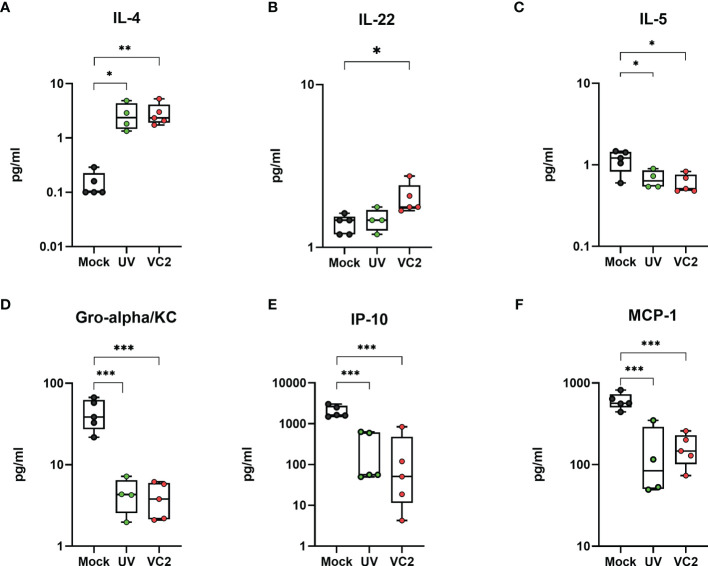
Pro-inflammatory and anti-inflammatory cytokines and chemokines in whole eye tissues. Cytokine IL-4, IL-22 and IL-5 **(A–C)**, and chemokine Gro-alpha/KC, IP-10 and MCP-1 **(D–F)** were measured from homogenized eye samples using the 26 plex immunoassay kit. *<P=0.05, **<P=0.005, ***<P=0.001 using One-Way ANOVA, n=5/group.

To address the status of immune response, we used a 26 plex immunoassay to detect Th1/Th2Th9/Th17Th22 and Treg associated cytokines and chemokines in homogenized eye samples at 5 DPI. The VC2 and UV-VC2 vaccinated animals exhibited a unique cytokine and chemokine expression profile compared to the mock-vaccinated animals. Specifically, the VC2 and UV-VC2 immunized animals had significantly higher IL-4 and IL-22 levels in the eye (**Figures 5A, B**). In contrast, the pro-inflammatory cytokine IL-5 and chemokine Gro-alpha/KC, IP-10 and MCP-1 was detected at lower levels in VC2 and UV-VC2 immunized animals compared to mock-vaccinated animals (**Figures 5C–F**). It has been reported that these pro-inflammatory cytokines are associated with tissue damage, neutrophil accumulation and increased severity of HSV-1 infection (47, 50). Overall, this data suggests VC2 immunization generates a unique adaptive response that reduces pro-inflammatory signals and that this reduction is associated with infiltrating γδT cells.

### Infiltrating γδT Cells Do Not Originate From an HSV-1 Specific Memory Population

**Figure 6:**
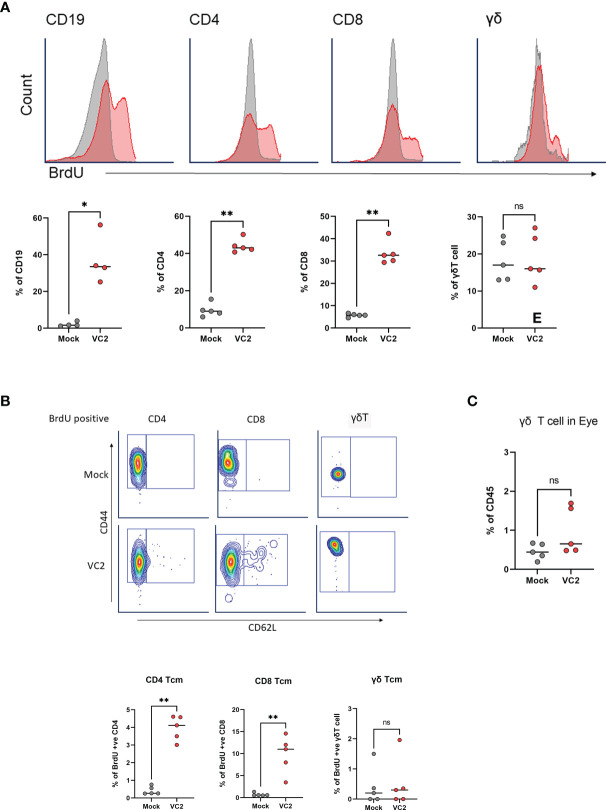
Analysis of antigen-specific memory T cell expansion in LN at 5DPI. **(A)** Representative histogram (top panel) and percentage of BrdU incorporation in CD19, CD4, CD8 and γδT cells in mLN cells using flow cytometry (bottom panel). **(B)** Representative gating (Top panel) and percentage of BrdU-positive TCM in CD4, CD8 and γδT cell populations. **(C)** Percentage of BrdU-positive γδT cell in the eye following infection. *<P=0.05, **<P=0.005 using Mann-Whitney test, n=5/group. ns, Non-Significant.

Several studies suggested that γδT cells may contain memory populations like αβT cells and can undergo memory-like expansion following antigen recognition (51–56). To address the possibility of whether infiltrating γδT cells represent an HSV-1 specific memory population, we performed a BrdU proliferation experiment *in-vivo*. Both mock and VC2-vaccinated animals were administered 1 ml concentrated BrdU per 100 g body weight *via* the intraperitoneal route one day before infection and continued every day. Following euthanasia, both eyes were removed and mLN cells were stained for BrdU positive cells. VC2-vaccinated animals exhibited marked incorporation (red) of BrdU in B (CD19), CD4 and CD8 T cells in mLN at 5DPI compared to mock-vaccinated (gray) animals, suggesting a pre-existing memory population for these cells (**Figure 6A**). However, there was no significant difference detected for BrdU-positive γδT cells in mLN tissues (**Figure 6A**, right panel), indicating the absence of a memory population. In addition, BrdU positive γδT cells in the vaccinated animals did not have a higher frequency of T central memory (TCM CD44+CD62L) as BrdU positive CD4+ and CD8+ T cells (**Figure 6B**) suggesting the absence of TCM in the proliferating γδT cell population. Further, there was no difference in BrdU-positive γδT cell in the ocular mucosa tissues following infection and only a small percentage of γδT were BrdU-positive in both mock and VC2-vaccinated groups (**Figure 6C**) suggesting infiltration of pre-existing rather than newly proliferative γδT cells in the eye following infection. Taken together, our data strongly suggest that the infiltrating γδT cells in vaccinated animals were not HSV-1 specific/experienced memory populations, but rather non-specific γδT cells recruited in the infected cornea in vaccinated animals with increased frequency.

### γδT Cell Infiltration Is Required to Control Ocular Immunopathogenesis

**Figure 7:**
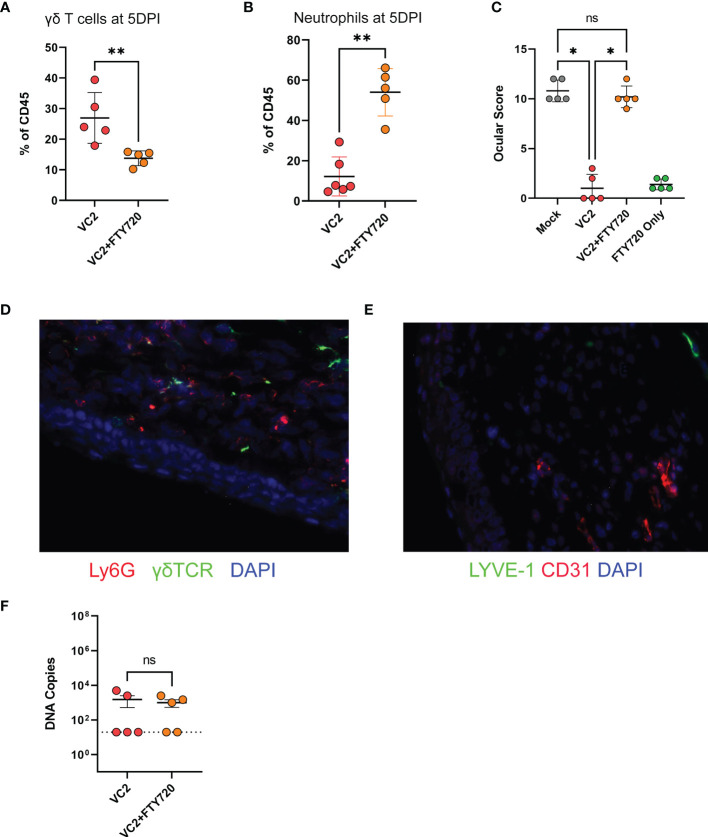
Ocular infiltration and score following inhibition of T cell migration. T cell migration in mouse eyes was inhibited by the administration of FTY720 on ocular surfaces. **(A)** γδT cell infiltration. **(B)** Neutrophil infiltration at 5DPI. **(C)** Ocular score following infection. **(D)** IFM on a representative OCT section for detection of γδT and neutrophil cell infiltration in ocular tissues. **(E)** IFM for CD31 and LYVE-1 expression. **(F)** Viral DNA copies in TG using qPCR. *<P=0.05, **<P=0.005 using the Mann-Whitney test, n=5/group. ns, Non-Significant.

Although it has been reported that γδT cells have an important role on the mucosal surface (57–60), the role of the γδT cells in vaccine-induced protection against herpes ocular immunopathogenesis and specifically herpes keratitis has not been investigated. The association of γδT cell infiltration (**Figure 3K**) and lower ocular damage (**Figure 1E**) suggest a functional role of this infiltrating population on corneal pathology following infection. This raises the question of whether the presence of this population is necessary for the control of HSV-1 induced keratitis in vaccinated animals. Unfortunately, there is neither a γδT cell KO animal in the Balb/C background nor an appropriate depleting antibody to remove this cell type from systemic and peripheral circulation to study the specific role of γδT cells following HSV-1 challenge. To circumvent this issue, we used FTY720 (Fingolimod, Sigma), an FDA-approved drug for immune suppression, which inhibits lymphocyte egress from both thymus and secondary lymphoid organs (61). Following infection in vaccinated animals, 1 drop (approximately 10 μl) of FTY720 (10mg/ml) was applied twice daily topically to the mouse eyes to prevent T cell infiltration. Because FTY720 was applied directly to the ocular surface, it was unlikely to alter cellular migration in other tissues. As expected, the FTY720 treatment lowered γδT cell infiltration at 5 DPI (**Figure 7A**). At the same time, FTY720-treated vaccinated animals exhibited a significant increase in neutrophil infiltration (**Figure 7B**) and concomitant increased ocular disease score (**Figure 7C**) compared to the PBS-treated and vaccinated animals. To confirm that FTY720 treatment did not increase ocular scores, naïve animals were also treated with FTY720 and showed minimal ocular damage in the absence of infection (**Figure 7C**, green). Further, there was no substantial increase in viral shedding in VC2-vaccinated animals compared to PBS-treated animals (data not shown) suggesting that the observed ocular damage is not due to persistent viral replication, but rather the result of increased neutrophil infiltration. The ocular damage persisted (gross observations) as long as the FTY720 treatment continued, and animals recovered quickly after FTY720 treatment termination (data not shown). Increased neutrophil infiltration (**Figure 7D**) and neovascularization (**Figure 7E**) was also detected more frequently after the challenge in FTY720-treated vaccinated animals. Although FTY720 treatment following challenge in vaccinated animals increased ocular pathogenesis, it did not have any effect on survival (data not shown), as well as on viral loads in the TG tissues (**Figure 7F**) suggesting that the role of γδT cell accumulation is limited to control of ocular immunopathogenesis induced by HSV-1 infection.

## Discussion

An over-active immune system during HSV-1 infection is generally thought to be the principal cause of corneal tissue damage (6). This damage is attributed to the influx of neutrophils (40, 41) and CD4 T cells that lead to visual impairment (4, 62). However, these cell populations have also been shown to be beneficial since their depletion rendered the animals more susceptible to infection. Thus, a balanced immune response is required to protect ocular tissues from immune-mediated damage, while ensuring clearance of the viral infection. We recently showed that the live-attenuated VC2 vaccine, which cannot enter neuronal axons and establish latency, protects mice against lethal ocular HSV-1 challenge and the development of HK (18). Herein, we show that VC2 intramuscular vaccination of mice results in γδT cell enhanced accumulation and reduction of infiltration neutrophils that results in substantial reduction of HSV ocular immunopathogenesis.

γδ T cells are known to participate in innate and adaptive immune responses (63–65). These cells can respond quickly to bacterial and viral infections because they can be activated by cytokines and toll-like receptor (TLR) signals without the need for T cell antigen receptor (TCR) activation (64, 66–68). This dual nature of γδ T cell biology is due to their non-MHC-restricted antigenic specificity enabling them to respond to a variety of cellular stress signals (69, 70). γδ T cells are present in lymphoid tissues and the blood in adult humans and rodents at low frequencies; however, they are enriched in epithelial and mucosal tissues. Tissue-specific γδ T cells are differentiated in various T cell subsets possessing specific functions acting as sensors of invading pathogens (71–74). In addition, a number of cytokines and chemokines are secreted by γδ T cells that can affect overall immune responses and tissue repair and healing (75). Several reports suggest that γδT cells are involved in mucosal immunity against several pathogens, although the mechanism may vary widely (51, 57–60, 76, 77). Previously, the presence of γδ T cells in the cornea of naïve mice infected ocularly with virulent HSV-1 was shown to be essential for protection against viral infection and resultant immunopathogenesis. Protection was associated with infiltration of γδ T CCR6 positive cells from the lymphatic system (78, 79). Our results agree with these findings. Specifically, we found that the γδ T cell population was prominent in mock-vaccinated animals. However, we observed a drastic increase of γδ T cell infiltration into the infected corneas as the result of VC2 intramuscular vaccination in comparison to mock-immunized animals suggesting that VC2 intramuscular immunization significantly altered the chemotactic movement of these cells from the lymphatic system into the infected corneas resulting in protection against virus-induced immunopathogenesis.

Several reports suggest that γδT cells are involved in mucosal immunity and exhibit cytotoxic, tissue repair, and regulatory functions (59, 60, 77, 80–83). Specifically, several studies found that γδT cells are involved in protection during ocular damage (60, 84–88). FTY720 mediated inhibition of γδT cell accumulation in the corneas of vaccinated mice significantly increased virus-induced immunopathogenesis. This suggests that the observed γδT cell accumulation is necessary to control exacerbated immune cell cytotoxicity, although the exact mechanism is not clear at present. It is worth noting that the reduction of neutrophil infiltration into the corneas of VC2-vaccinated animals was associated with the concurrent increase of γδT cells, since the absolute number of neutrophils was lower in vaccinated *versus* mock-vaccinated animals (**Figure 3F**). In addition, neutrophil numbers increased in the absence of γδT cells when VC2-vaccinated animals were treated with FTY720. This result suggests that γδT cells reduce tissue damage by inhibiting the recruitment of neutrophils into the infected corneal tissues from the systemic circulation. The expression of several cytokines was noted to be drastically different in VC2-vaccinated animals characterized by significant increases of IL-4, IL-22, and reduction of the inflammatory cytokine IL-5. Although γδT cells have been reported to secrete mainly IFNγ and IL-17, there are reports that these cells can also be a source of IL-4 (89, 90) and IL-22 (85). Both IL-4 and Il-22 were found to be involved in tissue repair (91–93). Future research should determine if γδT cells are the source of these cytokines and whether they are involved in tissue repair during HK.

Several studies proposed the presence of memory phenotypes in γδT cell populations and their expansion following infection (51–56, 81, 94). However, the BrdU proliferation assay (**Figure 6**) suggests that the γδT cell accumulation in the cornea did not represent proliferation of tissue-specific cells, but infiltration of cells originating from proximal lymph nodes. We hypothesize that VC2 induces HSV-1-specific tissue-resident memory (TRM) cells on the cornea of vaccinated animals, which recognize HSV-1 following infection and recruit γδT cells from the lymphatic system. It has been reported that gB498–505 epitope-specific TRM cells with CD73^+^CD8^+^ phenotype accumulated in mouse eyes following low-dose HSV-1 infection (95). We noted that uninfected Balb/CJ mice had a very limited number of T cells residing in their corneas including both γδT cell positive and negative populations. Tissue-resident-memory CD8+ T cells are known to bridge innate immune responses in neighboring cells and may be responsible for the observed γδT cell accumulation. Alternatively, there may be a HSV-1 specific γδ-TRM population residing in corneas that can efficiently recognize HSV-1 infection and recruit more γδT cells at the site of infection. Both cell types may exist at low abundance, rendering difficult an assessment of their phenotypic and functional properties.

Overall, our results strongly suggest that intramuscular immunization of mice with the live-attenuated VC2 vaccine strain significantly alters the infiltration of γδ T cells in the corneas of ocularly-challenged mice. These results suggest that a vaccine-generated tissue-specific memory response results in significant protection against HSV-1 immunopathogenesis. The mechanism by which VC2-intramuscular immunization results into the observed tissue-specific response is currently under investigation.

## Data Availability Statement

The raw data supporting the conclusions of this article will be made available by the authors, without undue reservation.

## Ethics Statement

The animal study was reviewed and approved by the Institutional Animal Care and Use Committee (IACUC), Louisiana State University, School of Veterinary Medicine.

## Author Contributions

RN and KK formulated and designed the study, analyzed and interpreted the data, and led the writing of the manuscript. AL evaluated all ocular infection results. VC constructed and verified all viruses used in this study. TC participated in the titration of different viruses and in viral neutralization experiments. RN and TC performed statistical analysis. All authors contributed to the article, critically reviewed and approved the submitted version.

## Funding

The work was supported by funds of the LSU Division of Biotechnology & Molecular Medicine (BioMMED), School of Veterinary Medicine, by a Governor’s Biotechnology Initiative grant from the Louisiana Board of Regents (to KK), and by Cores of the Center for Experimental Infectious Disease Research (CEIDR) and Louisiana Biomedical Research Network (LBRN) supported by NIH: NIGMS P30GM110670 and P20GM103424, respectively.

## Conflict of Interest

Louisiana State University has licensed the VC2 vaccine for genital herpes to Rational Vaccines. KGK serves as a consultant for Rational Vaccines, Inc.

## Publisher’s Note

All claims expressed in this article are solely those of the authors and do not necessarily represent those of their affiliated organizations, or those of the publisher, the editors and the reviewers. Any product that may be evaluated in this article, or claim that may be made by its manufacturer, is not guaranteed or endorsed by the publisher.

## Supplementary Material

Supplementary Figure 1 Gating strategy.
