# Supplementary figures and images for: Intramuscular Vaccination With the HSV-1(VC2) Live-Attenuated Vaccine Strain Confers Protection Against Viral Ocular Immunopathogenesis Associated With γδT Cell Intracorneal Infiltration
Source: Front Immunol. 2021 Nov 15;12:789454. doi: 10.3389/fimmu.2021.789454 (PMC8634438; doi:10.3389/fimmu.2021.789454)

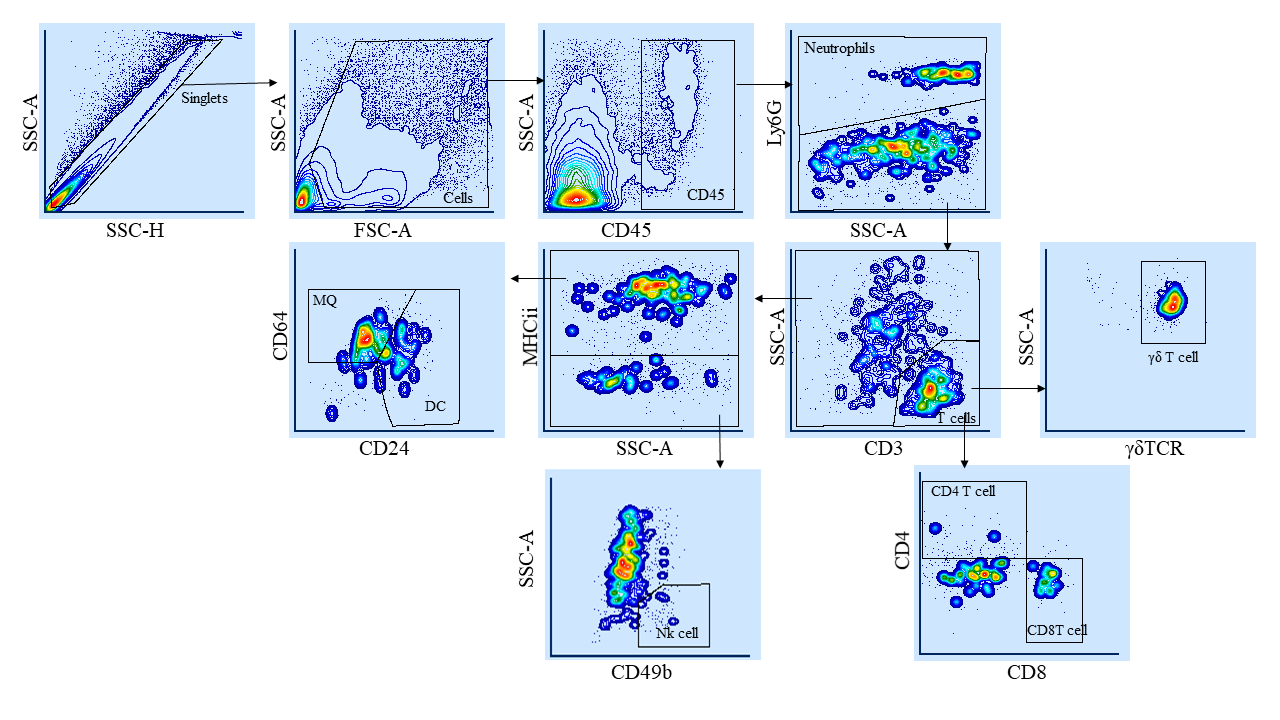

Supplement: Supplementary Figure 1 — Gating strategy. [file Image_1.tif]
